# Neuroprotective Effects of Low-Intensity Pulsed Ultrasound in Chronic Traumatic Encephalopathy Induced by Repetitive Head Collisions: A Narrative Review

**DOI:** 10.3390/biology14091148

**Published:** 2025-08-30

**Authors:** Min Zhang, Liang Zhao, Paulo Cesar Lock Silveira

**Affiliations:** 1 College of Sports and Health, Shandong Sport University, Jinan 250102, China; zhangmin@sdpei.edu.cn; 2 Office of Human Resources, Shandong Sport University, Jinan 250102, China; 3 Laboratory of Experimental Physiopathology, Program of Postgraduate in Science of Health, Universidade do Extremo Sul Catarinense, Av. Universitária, 1105 Universitário—Block S, Room 17, Criciúma 88806-000, SC, Brazil; psilveira@unesc.net

**Keywords:** chronic traumatic encephalopathy, LIPUS, head collision, football, contact sports, neurodegeneration

## Abstract

The repeated head impacts experienced by athletes have attracted significant interest from both the public and the scientific community; however, the neurobiological effects following the games are not well understood. For example, a single football match carries the risk of repeated concussive and subconcussive head impacts, which can increase the risk of developing neurodegenerative diseases. Chronic traumatic encephalopathy (CTE) is one of the neurodegenerative conditions athletes often face or are unaware of. However, addressing the disease progression in CTE is difficult to determine due to several reasons, such as the failure to identify risk factors, difficulty in differentiating CTE from other neurodegenerative diseases, and the lack of a specific mechanism by which CTE leads to tau protein accumulation. In addition, CTE symptoms overlap with other neurodegenerative conditions, such as Alzheimer’s disease (AD) and Parkinson’s disease (PD), which poses a challenge to producing specific targeted therapy. In this case, ultrasound represents a promising non-invasive technique that enables clear visualization of brain structures and may modulate neuronal activity. The term ultrasound encompasses various modalities; for example, high-intensity focused ultrasound (HIFU) employs thermal energy to ablate cells, whereas low-intensity pulsed ultrasound (LIPUS) delivers mechanical energy that activates molecular signaling pathways to impede the progression of CTE. Therefore, the LIPUS application could potentially minimize the risk of damage in the surrounding tissues of the brain and reduce the disease progression in individuals with CTE. Nevertheless, limited studies have been reported in the literature, with a poor mechanistic approach. Hence, this review aims to highlight the molecular signaling pathways, such as AKT, MAPK, and ERK, affected by LIPUS and emphasize the need for additional research to clarify its mechanistic effects in CTE management. Ultimately, this review aims to contribute to a nuanced understanding of LIPUS as a therapeutic strategy in addressing the complexities of CTE and its associated neurodegenerative disorders.

## Simple Summary

Sports collisions significantly contribute to the increase in neurodegenerative diseases. Chronic traumatic encephalopathy (CTE) is one of the common neurodegenerative conditions athletes often face in contact sports, with no cure, because the symptoms are similar to other conditions like AD and PD. Early diagnosis can be difficult, which may cause the condition to worsen. Additionally, this can affect participants’ quality of life and public health in various ways, including an increased risk of injuries and the cost linked to treatment, mental health, and long-term health consequences. Therefore, this review aims to highlight the possible molecular targets that can be regulated during the application of low-intensity pulsed ultrasound (LIPUS) for improving CTE.

## 1. Introduction

Concussion is a significant concern in contact and collision sports, including football, motor racing, equestrian activities, and other athletic competitions. Adjusting playing rules and enhancing guidelines for prompt concussion assessment could facilitate a safer return to play [1]. Sports involving high speed and force often cause head impacts. Factors such as the type of sport, the player’s position, and individual playing style can contribute to increased frequency and severity of head impacts. A sudden impact from speed and force in specific sports can cause the brain to move rapidly within the skull, leading to a concussion. A concussion is classified as a type of traumatic brain injury (TBI), which refers to brain damage caused by external mechanical forces [2]. Subconcussion, also caused by head impacts, may not produce noticeable symptoms like concussions but can still result in brain trauma [2]. Both concussions and subconcussions can raise the risk of developing chronic traumatic encephalopathy (CTE) when players are exposed to repeated head impacts. CTE is a neurodegenerative disease most frequently identified in athletes participating in contact sports such as football. It is characterized by memory loss, behavioral disturbances, and progressive cognitive decline [1]. A definitive diagnosis of CTE currently requires post-mortem examination of brain tissue. Clinical diagnostic criteria for CTE in living individuals remain under debate and consensus has not been reached. The prevalence of CTE is currently unknown. Recent research suggests that reported cases of CTE may be increasing, potentially due to greater awareness and improved diagnostic techniques. Evidenced from athletes who participate in collision sports, including football and boxing, shows decreased cortical thickness in the brain, indicating alterations of gray matter volume, gyrification, higher sulcal depth, decreased localized activity of neurons, and changes in the neural signals [1,2,3,4].

The repeated strikes to the head that induce CTE were first reported in 1928 by Martland during a match [5]. This condition was later termed “dementia pugilistica” and is now called CTE. Although the concept was established several decades ago, the pathophysiology of this neurodegenerative disease following a concussion remains unclear. The literature has reported that axonal disturbance is one of the primary consequences of CTE, which further exacerbates the metabolic abnormalities in neurons [6]. Thus, the hyperphosphorylation of 7 (p-tau) contributes to the aggregation of p-tau and, consequently, to disease progression [7]. Studies have shown that tau modification, particularly the accumulation of p-tau, represents a hallmark neuropathological feature of CTE rather than a direct clinical symptom [4,7,8]. Elevated p-tau levels serve as a key diagnostic criterion for CTE, although they do not solely account for disease progression [4]. However, the mechanisms by which tau modification contributes to CTE pathogenesis remain under investigation. Possible mechanisms are redox imbalance and calcium dyshomeostasis, which can lead to mitochondrial dysfunction and cell death [8,9]. Studies have observed, in case reports of several football players, cognitive impairments and neurotrauma following a concussion [10,11]. However, timely treatment and proper diagnosis are often unsuccessful because the individual may experience symptoms decades later, which increases the risk of developing CTE [9]. Therefore, exploring targeted therapy in most cases could not decrease the burden of disability due to CTE [1]. Moreover, there is no medication currently available for alleviating the symptoms of CTE. In this case, focusing on the primary cause of injury events caused by repeated concussion that triggers secondary events at the cellular and molecular levels may provide a firm therapeutic possibility.

The pioneering work from Wood and Loomis in 1927 about the ultrasound effect on biological samples significantly advanced the development of ultrasound technology for medical imaging in the late 1950s and 1960s [12]. Since then, there has been great interest in extending its applications to include therapeutic interventions and surgical support, making it a pivotal tool in modern medicine. Low-intensity pulsed ultrasound (LIPUS) is a particular type of ultrasound that distributes low-intensity pulsed waves to targeted tissues. This may be achieved through delivering mechanical and thermal energy, resulting in non-invasive stimulation of cells and tissues that potentially alter cell behavior [13,14,15]. Therefore, LIPUS may be a promising strategy for treating several neurodegenerative conditions, including TBI and CTE. LIPUS influences neural tissues through both mechanical and thermal mechanisms, both occurring simultaneously but with varying intensities, which modulate the neuronal activity by affecting ion channels and membrane capacitance. LIPUS parameters balance thermal and mechanical effects by maintaining low intensity and a pulsed output, which reduces heat production and allows mechanical effects like acoustic streaming and cavitation to dominate, leading to alterations in the therapeutic effect, such as increased cell proliferation and accelerated tissue healing. In particular, thermal effects modulate neural function by altering voltage-gated sodium channels and affecting synaptic transmission. These changes can modify neuronal firing patterns [16]. For instance, elevated temperatures trigger neurotransmitter release and receptor binding, thereby enhancing neuronal communication [16]. Temperature changes also sensitize various ion channels, resulting in increased neuronal excitability [16]. Additionally, LIPUS-induced thermal effects alter nerve conduction latency [17]. Non-thermal effects, including acoustic radiation force, acoustic streaming, particle displacement, and cavitation, also influence the neural functions [17]. Among these, cavitation is the most commonly utilized non-thermal effect due to its ability to produce localized and controlled mechanical effects, especially in drug delivery for brain injury treatment [18]. Cavitation involves the formation and oscillation of gas bubbles, primarily used for treating soft tissue lesions. Both stable and transient cavitation states of microbubbles can induce vessel invagination and disrupt the blood–brain barrier through strong inertial effects such as jetting and shock-wave formation [19]. Stable cavitation involves non-destructive bubble oscillations and transient and inertial cavitation, referring to the same phenomenon, which involves violent bubble collapse that generates shockwaves, increased temperature, and pressure. Prolonged stable cavitation can further increase kinetic energy exposure to cells. In the context of CTE, cavitation enables the safe opening of the BBB for drug delivery. However, excessive cavitation may lead to tissue damage, such as hemorrhage and cell death, likely due to elevated oxidative stress [18]. Using a cavitation feedback controller can help prevent microhemorrhage by modulating acoustic power levels [19]. In contrast, transcranial ultrasound stimulation (TUS) with low-intensity ultrasound can induce neural stimulation without cavitation, primarily through the interaction of ultrasound wave kinetic energy with mechanosensitive ion channels. Consequently, both short- and long-term neuronal excitability and firing rates may be increased [20].

Therefore, the use of low intensity is a promising strategy for treating several conditions, including neurodegenerative diseases and brain trauma. In the context of brain diseases, the ultrasound-induced neuromodulatory effect was initially observed by Harvey in 1929, who reported that high-frequency sonic waves trigger neuromuscular activities in the heart muscle [14]. Since then, studies have reported that both high-intensity and low-intensity ultrasound positively influence neuronal cells. LIPUS can modulate neuronal activity without increasing the temperature, and the effects are similar to those of conventional methods, such as pharmacological approach used to impact the brain cells, including astrocytes and neurons, without causing any side effects [13]. For example, pharmacological agents lack specificity and affect multiple targets. This can produce adverse side effects, and many of the current drugs fail to penetrate the blood–brain barrier (BBB), which restricts those drugs, whereas LIPUS facilitates BBB opening and decreases the neurotoxicity by inhibiting 1-Methyl-4-phenylpyridinium (MPP+) in PD [21]. While electromagnetic waves, such as MRI, are useful, ultrasound provides additional benefits as a mechanical wave for brain imaging, likely because it can penetrate deeper into the tissues without causing harmful effects associated with ionizing radiation. LIPUS enhances neural modulation by delivering high-frequency mechanical waves to neural tissue. For example, a study has reported that LIPUS delivered to the bilateral hippocampus induces the FNDC5/irisin pathway for neural modulation to treat dementia in mice [22]. In addition, LIPUS has been shown to increase the neurotrophic factors in the astrocytes [23], modulate the immune functions to decrease inflammation in the microglia [24], and promote BBB opening [25]. Furthermore, studies have shown that LIPUS can modulate mechanosensitive ion channels, increase anti-inflammatory effects, and enhance BDNF signaling while decreasing pro-inflammatory responses by inhibiting rho-associated, coiled-coil-containing protein kinase 1/phosphorylated myosin light chain 2 (ROCK1/p-MLC2) [24,26]. However, the use of different protocols and parameters in various cells hindered the exploration of specific mechanisms, making it difficult to finalize the targeting pathways in these cells. Therefore, this review will aim to cover the understanding of LIPUS as a therapeutic strategy in addressing the complexities of CTE and its associated neurodegenerative disorders.

## 2. Effect of LIPUS on Regulating Neuroinflammation in CTE

Neuroinflammation plays a crucial role in the development of CTE. LIPUS has been shown to reduce factors that contribute to neuroinflammation in CTE by interfering with the pro-inflammatory responses in resident glial cells, such as microglia and astrocytes, and regulating the recruitment of peripheral immune cells [27]. For instance, microglial cells act as the first line of defense during contact sports; however, their activation can trigger a pro-inflammatory response that leads to brain damage and hinders the brain’s repair and recovery processes [1]. LIPUS exposure at 1.0 MHz, with a 5% duty cycle and a 1 Hz repetition frequency, can improve this situation by reducing brain edema and BBB disruption while enhancing the expression of the tight junction protein zonula occludens-1 (ZO-1) through the activation of protein kinase B (AKT) signaling pathways in mice with traumatic brain injury (TBI) [28]. Furthermore, LIPUS various stimulation for 20 min per day at the intensity of 30 mW/cm^2^ with a frequency of 1 MHz and 38 kHz, 250 mW/cm^2^, 20%, and 90 min, decreases the pro-inflammatory response at the cellular level by inhibiting various pro-inflammatory mediators, including interleukin-1β 1β (IL-1β), IL-6, IL-8, tumor necrosis factor alpha (TNF-α), and caveolin-1 (Cav1), by modulating the mitogen-activated protein kinase (MAPK), extracellular signal-regulated kinase (ERK), toll-like receptor 4 (TLR-4), and nuclear factor kappa-light-chain enhancer of activated B cells (NF-κB) signaling pathways [29,30,31]. LIPUS also regulates macrophage phenotypes with a repetitive frequency of 10 Hz, a 10% duty cycle, and a duration of 20 s, to prevent infiltration through the MAPK and NF-κB signaling pathways, which is essential for reducing progressive neurodegeneration [24,26]. In microglial cells, the MAPK and NF-κB signaling pathways are implicated in upregulating pro-inflammatory cytokines, which lead to neurodegeneration through the loss of neuronal signaling and activation of the complement system [24]. Additionally, microglia stimulate the release of NO, which can cause abnormal dilation of cerebral vessels, leading to increased brain swelling [24]. LIPUS modulates nitric oxide (NO) production [32], which may alleviate abnormal vessel dilation and brain swelling by remodeling cerebral vessels and enhancing their diameter and length [33]. Moreover, LIPUS applied at an intensity of 357 mW/cm^2^ can reduce oxidative damage and endoplasmic reticulum stress in motor neurons by modulating Ca^2+^ signaling, nuclear factor of activated T cells (NFAT), NF-κB, and AKT pathways [33] (Table 1). A potential mechanism for this effect is that LIPUS decreases oxidative stress across various brain regions, which may help balance the M1 to M2 phenotype of microglia in CTE, as evidenced by their migration during LIPUS application [24]. LIPUS also influences the functional integrity of the BBB in CTE, which can inhibit the exaggerated initial immune response and neural inflammation by facilitating the entry of anti-inflammatory drugs [24]. In contrast, BBB damage in CTE allows plasma proteins, such as fibrinogen, to enter the brain, triggering neuroinflammation and astrocyte scarring through transforming growth factor beta (TGF-β) activation [34,35]. However, achieving satisfactory clinical outcomes in CTE is often challenging, as inflammatory mediators can induce beneficial responses that promote neuron repair and regeneration. Inhibiting the initial immune response can complicate efforts to achieve better clinical results [36]. Additionally, LIPUS can reduce neuroinflammation by enhancing dendritic cell-derived exosomes, which contain anti-inflammatory microRNAs such as miRNA-16 and miRNA-21 [37]. In summary, LIPUS modulates neuroinflammation and emerging trends in CTE. Notably, the use of focused ultrasound disrupts the BBB to facilitate drug delivery, and using ultrasound, especially high-intensity focused ultrasound, triggers localized thermal effects and induces an immune response, which can potentially decrease neuroinflammation.

## 3. Effect of LIPUS on Improving Endothelial Cell Function in CTE

The dysfunction of vascular endothelial cells contributes to the pathogenesis of CTE by decreasing NO production, which in turn reduces endothelial-dependent dilation [49]. One potential mechanism for this scenario is the competitive inhibition of NO synthase (NOS) by arginase, resulting in reduced NO production [49]. LIPUS can improve this condition in CTE by enhancing the vascular system and promoting enzymatic fibrinolysis [38]. For instance, Suchkova reported that LIPUS at a frequency of 40 kHz and an intensity of 0.25 to 0.75 W/cm^2^ completely inhibited NOS in an ischemic model involving rabbits [38]. This inhibition occurs due to the shear stress produced by LIPUS at intensities ranging from 1.6 to 2.0 W/cm^2^, which increases both NO and intracellular Ca^2+^ levels in endothelial cells [28]. Additionally, caveolins, particularly cav-1 and cav-2, are implicated in the inhibition of NO within brain endothelial cells. Studies have indicated that cav-1 levels increase in several models of brain injury [50,51]; however, its precise role in improving CTE remains unclear. Cav-1 is known to regulate NO-mediated matrix metalloproteinases, such as matrix metalloproteinase-2 (MMP-2) and MMP-9, which can increase blood–brain barrier (BBB) permeability. Elevating Cav-1 levels may contribute to quicker recovery in CTE cases. A study by Shindo et al. demonstrated that LIPUS at a frequency of 4.90 kHz and an intensity range of 117–174 mW/cm^2^ upregulates cav-1, which promotes angiogenesis [39]. Cav-1 plays a crucial role in the beneficial effects of LIPUS by converting mechanical stimuli from LIPUS into activated intracellular signaling pathways. For example, LIPUS-induced cav-1 triggers the phosphorylation of Fyn, AKT, ERK1/2, and focal adhesion kinase (FAK), thereby enhancing angiogenesis [39]. Furthermore, research has shown that the overexpression of cav-1 in neurons improves motor functions and preserves memory in mice with brain trauma [52]. Moreover, LIPUS decreases oxidative stress in endothelial cells by activating the PI3K/AKT signaling pathway [53], which subsequently reduces neuroinflammation in these cells within the context of CTE. In summary, LIPUS improves endothelial functions in CTE by promoting NO and Ca^2+^ levels with an intensity of 1.6 to 2.0 W/cm^2^.

## 4. LIPUS Treatment on Tau Protein Modification in CTE—A Possible Molecular Interaction

**Figure 1:**
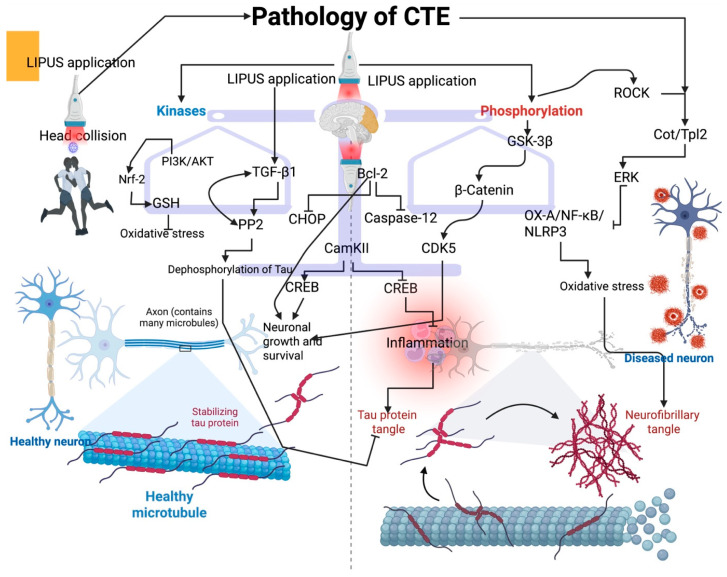
Effect of LIPUS on tau protein modification and possible molecular interventions. LIPUS application balances the kinases and phosphorylation to regulate tau modification. LIPUS triggers the ROCK/Cot/Tpl2 to reduce oxidative stress in CTE via ERK signaling. This can decrease the formation of neurofibrillary tangles. LIPUS induces GSK-3β to promote neuronal growth and survival via β-catenin and CDK5. LIPUS application in CTE activates the TGF-beta1, which in turn activates the PP2 to decrease the dephosphorylation of tau, and reduce the tau protein tangle. LIPUS can also activate the PI3K/AKT pathway to influence Nrf-2 signaling and promote GSH production, thereby reducing oxidative damage in the CTE brain. Additionally, LIPUS application triggers anti-apoptotic proteins, such as Bcl-2, which can inhibit CHOP and caspase-12. Furthermore, LIPUS can trigger the CamKII to induce CREB for neuronal growth and survival, whereas CamKII inhibits the CREB for triggering inflammation.

Although several studies detailed the role of tau protein function and its modification since its discovery in the 1990s, the role of tau in various health and disease conditions is unknown. LIPUS influences the balance of tau phosphorylation that prevents tau-mediated disease progression, including CTE [14]. Primarily, kinases and phosphatases can tightly regulate the levels of tau phosphorylation, and the disruption of kinase and phosphatase activities triggers tau-mediated pathology [54,55]. In this context, LIPUS can modulate these enzymes to balance the tau phosphorylation. For example, glycogen synthase kinase 3 beta (GSK-3β) phosphorylates the tau protein at different sites, such as Thr231 and Ser202, thus causing tau aggregation and pathological fibril formation [56] (Figure 1). Studies have shown that GSK-3β expression is increased in different tauopathies, including AD and CTE, and targeting GSK-3β could effectively reduce conditions like CTE [57]. LIPUS, at the intensity of 27.25 W/cm^2^ for 10 min per day for five days, can phosphorylate GSK-3β, thereby inhibiting it [58]. This can balance this scenario. Studies have shown that GSK-3β plays a crucial role in inducing tauopathies in CTE by phosphorylating tau residues, such as Thr231 and Thr202, in vitro [58,59,60,61]. Additionally, animal studies show that an increase in GSK-3β is linked to the TBI of animal models, indicating the role of GSK-3β in CTE pathophysiology [61]. MAPK families, such as ERK, phosphorylate tau, primarily through an oxidative stress-mediated mechanism, and their activity is shown to increase in CTE [62,63]. LIPUS, with an intensity of 30 milliwatts/cm^2^, can modulate the ERK activities to regulate its downstream effects in various cellular processes by activating the ROCK pathway [64]. A study has reported that LIPUS at the setting of 500 Hz with an intensity of 518 mW/cm^2^ for 30 min per day for three consecutive days, can regulate the OX-A/NF-κB/NLRP3 pathway in TBI via influencing ERK signaling, using 30 min of LIPUS application for three consecutive days in the TBI rat model [19]. JNK is another MAPK family that is dysregulated in the tauopathies. For example, the increase in JNK is observed in damaged axons, neurons, and astrocytes following TBI [65]. The elevation of JNK within the brain cells increases the cellular damage following TBI [66]. LIPUS, with an intensity of 528 mW/cm for 27 days, can modulate the c-Jun N-terminal kinase (JNK) signaling pathway to increase or decrease cellular proliferation and apoptosis via a LIPUS-mediated thrombin mechanism, thereby ameliorating glia-mediated inflammation and neuronal damage in a mouse model [42]. Cyclin-dependent kinase 5 (CDK5) is a crucial kinase enzyme that mediates neuronal damage and neuroprotection [67]. Dysregulation of CDK5 activity exacerbates injury progression in the brain of the TBI mouse model, and targeting CDK5 could be a potential candidate for ameliorating TBI [66]. LIPUS treatment at the intensity of 27.25 W/cm^2^ for 10 min in an in vitro study regulated the CDK5 pathway through the GSK-3β/β-catenin pathway, promoting neural growth by triggering axon outgrowth in vitro [58]. The overexpression of dual-specificity tyrosine phosphorylation-regulated kinase 1A (DYRK1A) may potentially increase tau phosphorylation at the various sites of tau residues, such as Thr-212, Thr-205, and Thr-231 [67,68,69,70], following brain injury and neurodegeneration [71]. Although no studies have been reported on the involvement of LIPUS in modifying DYRK1A in TBI, including CTE, LIPUS can downregulate DYRK1A expression by targeting miRNAs, such as miR-192-5p, in cerebral brain injury [71]. Calcium/Calmodulin-Dependent Protein Kinase II (CaMKII) can be involved in triggering both neurotoxic and neuroprotective effects, depending on the timing of the injury [72]. A study has shown that LIPUS (15 min of 1 MHz LIPUS) can modulate neuronal activity in the hippocampal region of rats by targeting the CaMKII-CREB pathway within 30 min of LIPUS exposure [41]. Moreover, LIPUS can also modulate PKA signaling in in vitro studies, and the increase in PKA is linked to tauopathy [73]. A possible mechanism is the cAMP/PKA/NLRP3 pathway that LIPUS can target following TBI [74].

In contrast to kinase activity, activating the dephosphorylation process may effectively reduce the tauopathy [42]. For example, protein phosphatase 2A (PP2A) is the major phosphatase that dephosphorylates tau and decreases tauopathy; activating PP2A may facilitate rapid recovery from TBI [75]. Although there is no direct evidence of LIPUS in activating PP2A, it has been reported that LIPUS effectively modifies various cellular processes by influencing the signaling pathways, and it is plausible that PP2A may be involved in mediating some of these cellular processes during LIPUS exposure in TBI and CTE. Calcineurin is another protein phosphatase that dephosphorylates tau at various sites, including T231 and S262 [76]. Dysregulation of calcineurin influences the dendritic remodeling following TBI, which can further develop neuronal damage. For example, calcineurin subunits (CnA and CnB) are altered in the hippocampus, affecting neuronal excitability and plasticity [77]. A study has shown that LIPUS exposure at the intensity of 357 mW/cm^2^ on days 2 and 4 increases the Ca^2+^-dependent signaling, including calcineurin/NFAT signaling, which improves the morphological maturation and proliferation of motor neuron cells [33].

## 5. Effect of LIPUS on Reducing Oxidative Stress in CTE

Oxidative stress exacerbates the TBI and CTE conditions and thus worsens the clinical outcomes. Activating nuclear factor erythroid 2-related factor 2 (Nrf2) can play a protective role in CTE [78]. A study has reported that LIPUS can activate Nrf2 by triggering the PI3K/Akt pathway, thus mitigating oxidative stress [79]. Additionally, CTE leads to an increase in iron accumulation in the brain, which in turn induces lipid peroxidation-mediated ferroptosis [80]. LIPUS activates pathways, such as the Nrf2/Keap1/HO-1, to mitigate oxidative stress and ferroptosis [73], by affecting glutathione (GSH)-related processes with an intensity of 60 mW/cm^2^ with a 50% duty cycle [81]. For example, the inhibition of SOD2 expression induces the oxidative stress-mediated tau phosphorylation at the sites of Thr205 and Ser396 in neural cells [82,83], and this can be reversed with the LIPUS-induced GSH activation [82,83]. ER stress and the subsequent unfolded protein response (UPR) play a crucial role in the progression of secondary brain injury, including inflammation, ischemia, and excitotoxicity, which ultimately triggers neuronal death. LIPUS can regulate this scenario by triggering Ca^2+^ signaling, which enhances antiapoptotic pathways like BCL-2, while regulating proapoptotic proteins such as CCAAT/enhancer-binding protein-homologous protein (CHOP) and caspase-12. This reduces the ER stress, oxidative stress, and mitochondrial dysfunction, thereby increasing the survival of neurons [33]. Moreover, LIPUS activates the pERK signaling and its downstream eIF2α to promote neuronal survival at the intensity of 500 mW/cm^2^ [84]. Other signaling pathways, such as the BDNF/CaMKII/Akt signaling pathway, can also be activated by LIPUS to reduce oxidative damage in brain injury [84]. Furthermore, oxidative stress-induced activation of tau-related kinases affects tau phosphorylation, and LIPUS can influence these kinases, primarily MAPKs, p38, JNK, and ERK, in CTE [83].

## 6. The Effect of LIPUS in Drug Delivery for Improving Neuroinflammation in CTE

**Figure 2:**
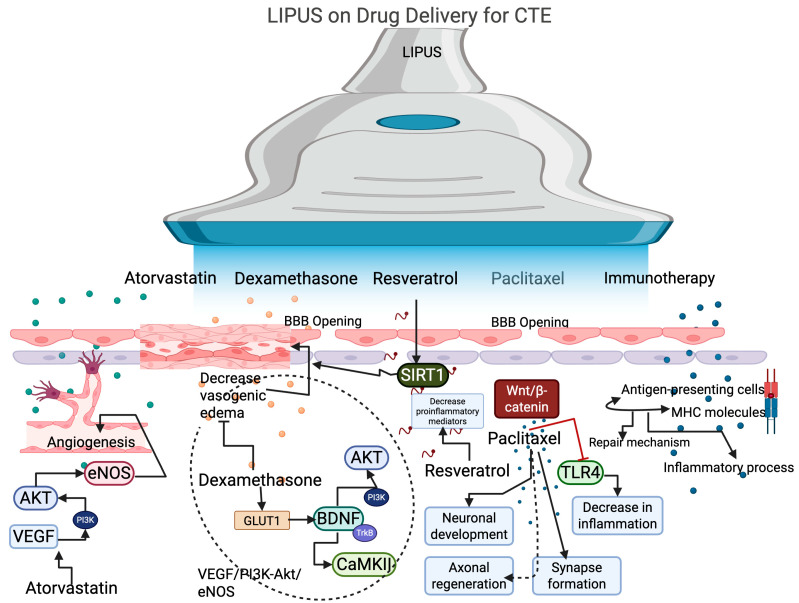
The effect of LIPUS on promoting drug delivery by opening the BBB. LIPUS with atorvastatin enhances angiogenesis in CTE by modulating the signaling pathways of VEGF, AKT, and eNOS. LIPUS with dexamethasone activates the GLUT1 signaling pathway, which further activates BDNF and its downstream targets, such as CaMKII and AKT, via TrkB and PI3K, thereby decreasing vasogenic edema. Resveratrol with LIPUS activates the SIRT signaling pathway to decrease edema and reduce pro-inflammatory mediators, thereby decreasing neuroinflammation. Paclitaxel activates the Wnt/beta-catenin to promote neuronal development, axonal regeneration, and synapse formation. Additionally, it can decrease TLR4 expression to reduce neural inflammation in CTE. Immunotherapy with LIPUS promotes the repair mechanism and decreases the inflammatory process.

The entry of drugs into the brain poses significant challenges, even with advancements in science. However, the application of LIPUS may help overcome these obstacles by dilating the BBB. For instance, a study demonstrated that LIPUS combined with microbubble administration temporarily enhances the opening of the BBB, thereby increasing the delivery of drugs [85]. Drugs such as temozolomide and irinotecan, which are used to treat glioblastoma, benefited from LIPUS-induced BBB opening, leading to improved drug delivery in a rabbit model [86]. Furthermore, research indicated that LIPUS enhances the delivery of immunotherapeutic agents, including antigen-presenting cells that attract T cell chemokines, in a preclinical glioma model at the frequency of 1 MHz with a duty cycle of 2.5% [25] (Figure 2). Although this study did not evaluate the impact of LIPUS on immunotherapy delivery in CTE conditions, effective delivery of these compounds could potentially enhance the repair processes in CTE. Additionally, paclitaxel has been shown to reduce brain injury from repeated head trauma in mice [87]. For example, LIPUS improves the delivery of paclitaxel across the BBB in a mouse model [88]. Dexamethasone is known to improve prognosis in TBI by reducing vasogenic edema [89]; however, its administration can lead to neurotoxicity due to mitochondrial impairment. In such cases, LIPUS has been shown to alleviate neurobehavioral deficits and brain injury caused by dexamethasone treatment by promoting GLUT1 and BDNF/CaMKII/Akt signaling pathways [84]. Atorvastatin has been found to improve clinical outcomes in TBI, and its combination with LIPUS enhances angiogenesis by upregulating VEGF and PI3K-Akt-eNOS signaling pathways, facilitating recovery from CTE [90]. Triptolide, known for its neuroprotective effects, can reduce brain injury, and a study has reported that focused ultrasound can enhance triptolide delivery to the brain by triggering BBB opening in mice [91]. However, the specific role of LIPUS in this context requires further exploration. Research has also shown that resveratrol treatment can alleviate TBI-related behavioral abnormalities by reducing edema and contusion volume and preserving hippocampal neurons [92]. Although no studies have investigated the effects of LIPUS in conjunction with resveratrol, one study indicated that LIPUS combined with resveratrol inhibits ovarian cancer cell proliferation by inducing apoptosis [79].

## 7. Biological Mechanisms: From Animal Studies to Human Care

Small animal groups can provide valuable insights into how different ultrasound protocols might be translated to human care. For example, LIPUS improves the collagen content and mechanical strength in various injuries, such as tendon, ligament, and bone injuries, in many animal models [90,91]. These results were further replicated with the human trials in improving bone formation and decreasing healing time with a specific ultrasound signal [93,94,95]. Also, LIPUS treatment achieved better results on improving delayed unions, which can be referred to as fractures that failed to reveal radiographic progression between 3 and 9 months [96]. In terms of biological mechanisms, LIPUS influences all the phases of healing (inflammatory, reparative, and remodeling phases). For example, LIPUS induced “nano motion” boosts the deposition of collagen and aggrecan at the damaged site, along with improving biochemical response via integrins by converting biomechanical stimulation [73,97]. This can reconstitute the brain’s extracellular matrix. Another possible biological mechanism in brain trauma is that LIPUS can trigger the mechanical stress in the damaged site of the brain, which further improves neurogenesis, calcium uptake, and protein synthesis [98]. LIPUS alters the inflammatory response for improving microglial activation in cerebral hemorrhage in many animal models, and the possible molecular targets are PI3K/Akt-NF-κB, which decrease microglial inflammation [42,53]. However, these results have not been implemented in the human trials yet. Furthermore, LIPUS has been shown to improve the healing of many soft-tissue injuries. For example, LIPUS accelerates the healing process in the calcaneus tendon injuries in the rat model [99]. However, translating the results of animal studies to human care has several limitations. For example, extremities in humans possess more subcutaneous fat when compared to animals, and this increased amount of fat may attenuate the LIPUS waves significantly, lessening the effects of LIPUS on the targeted soft tissues [100]. In addition, results from animal studies have often focused on the effects of LIPUS in acute injuries, but the clinical trials often focus on chronic conditions, and the different injury stages may affect the LIPUS effects differently. Also, individual differences in healing capacity due to age, genetics, and other comorbidities may influence the injury response to LIPUS. Also, optimizing LIPUS parameters for each individual with different injury type and monitoring the accuracy of the ultrasound beam focal point in deeper tissues may be challenging in clinical practice. However, the frequencies below 100 kHz could be useful for structural targets, especially for deeper tissues, because low frequency, typically from 20 to 100 kHz, can penetrate deeper tissues when compared to higher frequencies. These results may be due to reduced energy loss, which allows the waves to travel longer and penetrate deeper tissues. At low duty cycles without microbubbles, LIPUS can induce mechanical effects that can trigger different cellular responses via mechanotransduction, resulting in the activation of specific signaling pathways. This can improve cell proliferation, differentiation, and specific gene expression to promote tissue repair and regeneration.

## 8. Clinical Applicability and Safety Profile

While LIPUS shows promise as a therapy for various brain conditions, including TBI and CTE [28,78], it is crucial to assess its clinical applicability and safety before further exploration. For instance, animal studies have demonstrated that LIPUS can promote angiogenesis and neurogenesis in the brain, but translating these findings into clinical trials is still in the early stages. A pilot study on AD indicated a reduction in cognitive decline [101]. However, this study had limitations, such as not measuring the cancellous and cortical bone volume ratio, which could affect ultrasound transmission [101]. Additionally, the safety of intracranial ultrasound diffuse reflection was not evaluated [101]. Consequently, these preliminary results need validation through more clinical research. Regarding safety, avoiding mechanical bioeffects, such as cavitation in targeted or nearby tissues, is essential to prevent adverse side effects. Elevated thermal effects, which can cause significant tissue damage—especially at high power and medium duty cycle settings—may raise temperatures to as high as 42 °C [102]. In this case, the mechanical effects can be a more promising strategy as thermal effects occur only after 40 °C. Developing reliable monitoring techniques to track LIPUS-induced changes in the brain will be valuable for treating CTE and other brain diseases.

## 9. Limitations of LIPUS Use

While LIPUS shows potential for treating brain injuries, several limitations should be considered before its use. Although the use of LIPUS is generally safe for therapeutic applications, excessive or prolonged exposure can result in tissue damage within the brain. Therefore, a more comprehensive understanding of LIPUS parameters, thermal effects, and cellular responses across different brain regions is necessary. Additionally, targeting specific regions with LIPUS can be challenging, as some brain areas are obstructed by intervening structures. The dense bone composition of the skull also restricts the penetration of ultrasound waves into the brain, potentially diminishing the effectiveness of LIPUS treatment. The presence of dense cranial bone may impede the effectiveness of LIPUS in treating CTE, as skull density can significantly affect the transmittance of ultrasound waves. Consequently, the design and clinical application of LIPUS must address the challenge posed by this cranial barrier. Recent research has identified optimal LIPUS treatment parameters for overcoming bone density and thickness in patients with AD, specifically a sound pressure below the probe of 1.3 MPa, a duty cycle of 5%, and a frequency of 0.5 MHz [101]. While some evidence suggests that LIPUS may increase bone density, its effects on axonal degeneration, cell death, and disruption of the BBB in relation to bone healing and density remain inadequately established. Nonetheless, LIPUS has been shown to enhance peripheral nerve regeneration and facilitate nerve recovery, which may help mitigate axonal degeneration [103]. In this case, using FUS allows localized brain treatment by facilitating the penetration of ultrasound beams with millimeter and millisecond resolutions to the specific brain regions [104]. This can minimize the effects of intervening tissues. However, the skull could distort the ultrasound waves, which can challenge the use of FUS, as it mispredicts the intensity of ultrasound waves that are delivered to the specific brain region. Furthermore, high intensities induce inertial cavitation, which can lead to vascular and tissue damage. Real-time control of cavitation is essential yet challenging. Methods such as feedback control, acoustic emissions, and power cavitation imaging help manage cavitation, but each has its own drawbacks [105,106]. Also, the effect of LIPUS on modifying the regional specificity of the brain with a precise mechanism is unknown, and the use of preclinical results in clinical translation faces challenges, such as safety concerns that require rigorous clinical trials [107]. Also, the use of FUS can only cover a small region of the brain, which is also a major limitation when targeting larger brain areas [108]. Other limitations, such as axonal degeneration, cell death, and the disruption of the BBB, are also associated with LIPUS therapy. Therefore, optimizing LIPUS protocols by adjusting factors like intensity, frequency, and pulse duration for specific brain injuries could help overcome these challenges.

## 10. Alternative Approaches to Treat CTE Beyond LIPUS

While there is no definitive cure for CTE, several alternative strategies have been explored beyond LIPUS. These include providing cognitive rehabilitation therapy, using medications such as antidepressants and anti-anxiety drugs to manage symptoms, and making lifestyle changes. Emerging therapies such as transcutaneous spinal cord stimulation, transcranial magnetic stimulation, and transcranial direct current stimulation are useful for neurostimulation, targeting specific brain regions. For example, epidural electrical cortical stimulation is a minimally invasive technique involving small electrodes inserted into the epidural space to selectively activate certain cortical areas [109]. Additionally, neurorestorative treatments like cellular cytokine solutions may enhance repair mechanisms by modulating neuroinflammatory processes. Animal models of TBI show significant reductions in MPO and improvements in motor recovery when neuroinflammation is altered [110]. Therapies such as stem cell factor and granulocytic colony-stimulating factor have demonstrated notable neuroprotective and neurorestorative effects in TBI [111]. For instance, bone marrow-derived mesenchymal stem cells (MSCs) have improved cognition, speech, and memory in chronic TBI patients without negative side effects [112]. Moreover, integrative approaches, such as combining drug therapy, occupational therapy, muscle therapy, and acupuncture, have substantially enhanced outcomes for TBI patients [108,113].

## 11. Conclusions

In conclusion, LIPUS presents a promising approach to enhancing drug delivery and addressing the challenges associated with CTE. LIPUS effectively prevents mitochondrial dysfunction and oxidative stress, promotes angiogenesis, mitigates ferroptosis, and reduces endoplasmic reticulum stress by facilitating the opening of the blood–brain barrier in CTE condition through activating various signaling pathways, such as GLUT1, BDNF/CaMKII/Akt, PI3K-Akt-eNOS, Nrf2/Keap1/HO-1, Ca^2+^ signaling, BCL-2 CCAAT/enhancer-binding protein-homologous protein (CHOP), and caspase-12. Additionally, its ability to modify the tau protein through distinct pathways, including GSK-3β, ERK, cAMP/PKA/NLRP3, and calcineurin/NFAT signaling pathways, further underscores its therapeutic potential. By employing a mechanistic approach and harnessing advanced technologies, LIPUS could serve as a valuable tool for improving outcomes in individuals affected by CTE resulting from sports-related collisions.

## Figures and Tables

**Table 1:** Application of LIPUS with various intensities on triggering molecular events in brain pathology.

LIPUS Parameter Intensity (mW/cm^2^)	Studies	Molecular Interventions	References
357 mW/cm^2^	NSC-34 cell line	LIPUS activate Ca^2+^, NFAT, NF-κB, and AKT signaling for reducing oxidative stress in the motor neuron	[33]
0.25 to 0.75 W/cm^2^	Rabit model	LIPUS inhibits the NOS	[38]
1.6 to 2.0 W/cm	Mice model with TBI	LIPUS increases NO and intracellular Ca^2+^ levels in endothelial cells	[28]
117–174 mW/cm^2^	Mice model	Upregulates cav-1 for promoting angiogenesis	[39]
255 mW/cm^2^, 10 min/d, 10 d	Rat model	Modulates the cilia of the rat	[40]
50 mW/cm^2^	Rat model	LIPUS influx the Ca^2+^ to regulate neuronal activity	[41]
528 mW/cm^2^	Mice model	LIPUS inhibits the PI3K/Akt-NF-κB signaling for reducing glial inflammation	[42]
15 W/cm^2^	Mice model	Opens calcium-permeable mechanosensitive ion channels, including TRPP1/2, TRPC1, and Piezo1 for neural modulation	[43]
69.3 mW/cm^2^	NSCs	Upregulates Notch1 and Hes1 to regulate proliferation and differentiation on neurons	[44]
360 mW/cm^2^	Rat model	Increases the c-Fos, dendritic spine density, and alters the GluN2A, GluN2B, and GluR1 via BDNF- mediated pathways	[45]
0.12 W/cm^2^	Rat model	Increases the axonal growth by activating netrin-1 and DCC in cortical neurons	[46]
<100 W/cm^2^	Mice model	Decreases IL-17A and Notch1 for protecting oligodendrocytes in neurons, repairs white matter, and improves recovery	[47]
70 and 165 mW/cm^2^	Mice model	Autophagy markers such as LC3BII/LC3BI increased with LIPUS in neurons	[48]

## Data Availability

Not applicable.

## Abbreviations

The following abbreviations are used in this manuscript:

CTE	Chronic traumatic encephalopathy
AD	Alzheimer’s disease
PD	Parkinson’s disease
LIPUS	Low-intensity pulsed ultrasound
BBB	Blood–brain barrier
FNDC5	Fibronectin type III domain-containing protein 5
ROCK1	rho-associated, coiled-coil-containing protein kinase 1
p-MLC2	Phosphorylated myosin light chain 2
ZO-1	Zonula occludens-1
AKT	Protein kinase B
IL	Interleukin
TNF-α	Tumor necrosis factor alpha
Cav.1	Caveolin-1
MAPK	Mitogen-activated protein kinase
ERK	Extracellular signal-regulated kinase
TLR-4	Toll-like receptor 4
NF-kB	Nuclear factor kappa-light-chain-enhancer of activated B cells
NO	Nitric oxide
TGF-β	Transforming growth factor beta
FAK	Focal adhesion kinase
GSK-3β	Glycogen synthase kinase 3 beta
JNK	c-Jun N-terminal kinase
CDK5	Cyclin-dependent kinase 5
DYRK1A	Dual-specificity tyrosine phosphorylation-regulated kinase 1A
CaMKII	Calcium/calmodulin-dependent protein kinase II
PP2A	Protein Phosphatase 2A
Nrf2	Nuclear factor erythroid 2-related factor 2
UPR	Unfolded protein response
CHOP	C/EBP homologous protein
BDNF	Brain-derived neurotrophic factor
GLUT1	Glucose transporter 1
VEGF	Vascular endothelial growth factor
eNOS	Endothelial nitric oxide synthase
eIF2α	Eukaryotic translation initiation factor 2
